# Pre-season football preparation in the era of COVID-19: Croatian Football Association Model

**DOI:** 10.7189/jogh.10.010352

**Published:** 2020-06

**Authors:** Dragan Primorac, Vid Matišić, Vilim Molnar, Zoran Bahtijarević, Ozren Polašek

**Affiliations:** 1 St. Catherine Specialty Hospital, Zagreb, Croatia; 2 University of Split School of Medicine, Split, Croatia; 3 Eberly College of Science, Penn State University, State College, Pennsylvania, USA; 4 The Henry C. Lee College of Criminal Justice and Forensic Sciences, University of New Haven, West Haven, Connecticut, USA; 5 University of Osijek School of Medicine, Osijek, Croatia; 6 University of Osijek Faculty of Dental Medicine and Health, Osijek, Croatia; 7 University of Rijeka School of Medicine, Rijeka, Croatia; 8 Medical School REGIOMED, Coburg, Germany; 9 Children Hospital Zagreb, Zagreb, Croatia

With over 3.3 million confirmed cases worldwide and over 230 000 confirmed deaths, the COVID-19 pandemic is, without doubt, the most important event of the 21^st^ century so far. The COVID-19 pandemic has upended all areas of life - and sports is no exception. Unsurprisingly, this pandemic has also stopped the sporting calendar, with professional leagues everywhere suspending their activities to limit the spread of the virus. Guidelines for COVID-19 diagnosis change on a day-to-day basis, as we progress with primary studies and gain more. The guidelines encompass clinical criteria, epidemiological patient history, and molecular diagnostics. Football coaches are keen on perspective and realize that the challenges they face now and in the months ahead hardly compare to the hardships the world confronts during a pandemic, but the return to pre-pandemic activities requires medical guidelines.

Croatian Football Federation, with its Medical Committee is launching a new model of pre-season systematic examination of football players with a particular emphasis on diagnosing COVID-19. Studies suggest that the proportion of asymptomatic cases ranges from 17.9% onboard the Diamond Princess cruise ship, up to 78% detected in newly reported Chinese data [1,2]. In our opinion, there is little doubt that COVID-19 is far more widely distributed than some may believe, knowing the data implies that an asymptomatic person can spread the infection, particularly during the incubation period [3-5]. Identifying the asymptomatic carriers of the disease has become crucial in preventing further spread of the epidemic.

**Figure:**
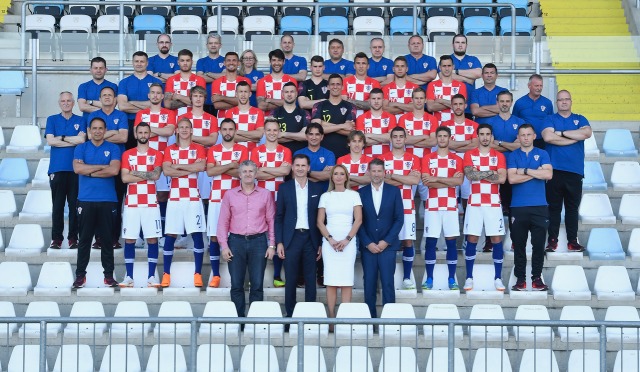
Photo: Croatian National Football Team before the 2018 FIFA World Cup with the management of its official health care organization, St. Catherine Hospital. Photo by Drago Sopta (used with permission).

It is expected that majority of people who recover from COVID-19 will not have long-term consequences. However, COVID-19 is a multi-organ disease commonly affecting the lung, heart, kidney, digestive tract, and nervous system, with unclear situation regarding long-term consequences [6,7]. Survivors of the severe COVID-19 disease were reported to have changes in their lungs, similar to those observed in SARS, marked by the diminished pulmonary capacity [8]. Important lessons on long term outcomes for patients who contracted COVID-19 are still to be learned, but in order to adapt to the global situation, we believe it is paramount to draw parallels with the epidemics of SARS (severe acute respiratory syndrome) and MERS (Middle East Respiratory Syndrome). A recent study showed that approximately 20% of COVID-19 patients suffered from cardiac injuries [9]. Studies of SARS and MERS reported high occurrence of hypertension, persistent tachycardia and myocarditis in convalescent patients. A study on 121 patients who were infected with SARS showed that hypertension occurred in over half of all the patients, while 71.9% of them developed persistent tachycardia [10]. One the other hand, it has been shown that MERS causes myocarditis, most likely by the direct viral infection; the same was implied for COVID-19 [11]. Acute kidney injury and proteinuria were also reported in COVID-19 patients, suggesting direct cellular damage of the kidney tissue [7]. By comparison, COVID-19 patients who were treated in the ICU, particularly those who were mechanically ventilated, were reported to suffer from “post-intensive care syndrome”, most likely because the lack of oxygen in blood [12]. All these reports suggest that COVID-19 infection might be regarded as higher risk than it was initially believed. This is of paramount importance in football activities, knowing that career development requires a lot of time and effort invested into prevention of injury, disease, disability and even death [13]. The sport community finds itself facing uncharted territories in both the wake and the aftermath of this pandemic. Therefore, we firmly believe that coordinated, well-communicated and transparent action is of utmost importance if we want the return to regular activities be safe for all stakeholders.

Although professional football players are generally a healthy population without chronic respiratory, cardiac, renal diseases, as well as other chronic conditions, we must take COVID-19 seriously and act accordingly before returning to football pitches. Underlying genetic factors must also be taken into account as they might be aggravated by COVID-19 causing their clinical manifestation. Together with the list of medical examinations that players must undergo in order to be eligible to participate in UEFA competitions, we hereby propose a model for screening professional football players returning to the field after the lift of the ban on all sport activities because of the COVID-19 pandemic. Besides pre-season physical examination (primarily 12-lead ECG, ECG, spirometry with bronchodilatation test, diffusing capacity of the lung for carbon monoxide (DLCO) test and fractional exhaled nitric oxide (FeNO) test) and medical examination defined by the UEFA Medical Regulations (for the next season), we are proposing that every football player from the Croatian first National League must have negative consecutive two RT-qPCR COVID-19 pharyngeal swabs over a 5-day interval. Such tests are targeting two regions of the viral nucleocapsid gene (N1 and N2) or RNA-dependent RNA polymerase (RdRP) and envelope (E) genes [14]. This is of special interest due to the long virus incubation – median incubation period for COVID-19 was estimated to approximately five days [15]. However, it has been noted that time from exposure to onset of infectiousness (latent period) may be shorter than the incubation period [5]. Therefore, it is essential to do two subsequent tests during the proposed period. In addition to the detection of viral genetic material, we will target the immune response of the athlete being screened, looking specifically for antibodies (IgM and IgG) against the virus or viral antigens. Those tests are less complex than molecular tests but since antibody responses to infection take days to weeks to be detectable, serologic tests will not be reliable among those with recent exposure to virus. However, antibodies detected by this test indicate that a person had an immune response to COVID-19, implying the infection was subclinical if the person was asymptomatic. Serologic tests could play an important role in establishing diagnosis, if the COVID-19 patient with late complications of disease is examined since RT-qPCR could produce false-negative results, presumably because of the low viral load [14]. In addition to limiting the potential of viral spread with the start of regular sport activities, the results of this screening protocol will allow us to estimate how many football players have been infected nationally. The results will also provide information on the percentage of Croatian football players who have not had COVID-19 and are still at risk of being infected.

We propose that football players need a gradual return to physical activities during four separate phases. The first phase includes training in a small group, while the second phase comprises training of the entire team. Consequently, players will start with the national leagues’ competition (phase three), while in phase four, the clubs will be joining international competitions. Ideally, the club’s prior international competition should provide for all registered players, certificates issued by the accredited laboratories that the players are negative for COVID-19. In addition to all these procedures mentioned above, special preventive recommendations will be given to the football players and other team members in addition to the above described screening program prior to the continuation of competitive matches. Those include the following: 1) Trainings must be performed outdoors. Entry into club rooms and other closed spaces are prohibited. 2) No more than 5 players can participate in training sessions at the same time. The personal distance must be met at all times with at least 5 m separating the players. Players must use own lanes for running and sprints, if this requirement can’t be met, the same lane may be used by more players, but they must keep a distance between each other of at least 40 m when sprinting. Headers are not allowed in training sessions due to close contact of the ball with orifices of the body. 3) The number of coaches in training sessions should be kept to a minimum. Apart from the coaches only a physio or a team doctor may be present in the session. Protective equipment is mandatory for the medical team when they are getting in close contact to a player. Protective equipment includes face masks (N95, FFP2 or FFP3), protective gloves and face shields. 4) Original, sealed plastic water bottles must be used and properly discarded after training. They may not be shared between players. 5) Players and coaches must come to the training grounds alone, in their own cars, wearing appropriate clothes. Changing and shower rooms will not be available for the athletes nor coaches. 6) After the training session both players and coaches must go to their homes using the same transport they came with. This should be done orderly whilst respecting the social distancing measures. We propose to implement these measures first in the Croatian First League. They will also be recommended to lower leagues when they start with training and competitions and will be adhered to until a broader relaxation of preventive measures is advised by the local authorities. We presented our model to the leadership of FIFA and UEFA, with the goal of sharing our knowledge and ideas, as well as synchronising the actions of all the members during the COVID-19 pandemic. We believe that adherence to the recommendations and testing of players will drastically reduce the risk of them being exposed to SARS-CoV-2 and other pathogens. In turn, it should allow a steady return to football we all know and love.
